# Brazilian guidelines for the management of brain-dead potential organ donors. The task force of the AMIB, ABTO, BRICNet, and the General Coordination of the National Transplant System

**DOI:** 10.1186/s13613-020-00787-0

**Published:** 2020-12-14

**Authors:** Glauco Adrieno Westphal, Caroline Cabral Robinson, Alexandre Biasi Cavalcanti, Anderson Ricardo Roman Gonçalves, Cátia Moreira Guterres, Cassiano Teixeira, Cinara Stein, Cristiano Augusto Franke, Daiana Barbosa da Silva, Daniela Ferreira Salomão Pontes, Diego Silva Leite Nunes, Edson Abdala, Felipe Dal-Pizzol, Fernando Augusto Bozza, Flávia Ribeiro Machado, Joel de Andrade, Luciane Nascimento Cruz, Luciano Cesar Pontes de Azevedo, Miriam Cristine Vahl Machado, Regis Goulart Rosa, Roberto Ceratti Manfro, Rosana Reis Nothen, Suzana Margareth Lobo, Tatiana Helena Rech, Thiago Lisboa, Verônica Colpani, Maicon Falavigna

**Affiliations:** 1 Hospital Moinhos de Vento (HMV), R. Ramiro Barcelos, 910, Porto Alegre, RS 90035000 Brazil; 2 Hospital Municipal São José (HMSJ), Joinville, SC Brazil; 3 Centro Hospitalar Unimed, Joinville, SC Brazil; 4 Hospital do Coração (HCor), R. Desembargador Eliseu Guilherme, 147, São Paulo, SP 04004030 Brazil; 5 Universidade da Região de Joinville (UNIVILLE), R. Paulo Malschitzki, 10, Joinville, SC 89219710 Brazil; 6 Clínica de Nefrologia de Joinville, R. Plácido Gomes, 370, Joinville, SC 89202-050 Brazil; 7 Hospital de Clínicas de Porto Alegre (HCPA), R. Ramiro Barcelos, 2350, Porto Alegre, RS 90035007 Brazil; 8 Universidade Federal de Ciências da Saúde de Porto Alegre (UFCSPA), Sarmento Leite, 245, Porto Alegre, RS 90050-170 Brazil; 9 Hospital de Pronto de Socorro (HPS), Porto Alegre, RS Brazil; 10 General Coordination Office of the National Transplant System, Brazilian Ministry of Health, Esplanada dos Ministérios, Bloco G, Edifício Sede, Brasília, DF 70058900 Brazil; 11 Faculdade de Medicina, Universidade de São Paulo (USP), Av. Dr, Arnaldo 455, Sala 3206, São Paulo, SP 01246903 Brazil; 12 Universidade do Extremo Sul Catarinense (UNESC), Av. Universitária, 1105, Criciúma, SC 88806000 Brazil; 13 Intensive Care Unit, Hospital São José, R. Cel. Pedro Benedet, 630, Criciúma, SC 88801-250 Brazil; 14 National Institute of Infectious Disease Evandro Chagas, Fundação Oswaldo Cruz (FIOCRUZ), Av. Brasil, 4365, Rio de Janeiro, RJ 21040360 Brazil; 15 Instituto D’Or de Pesquisa e Ensino (IDOR), R. Diniz Cordeiro, 30, Rio de Janeiro, RJ 22281100 Brazil; 16 Hospital São Paulo (HU), Universidade Federal de São Paulo (UNIFESP), R. Napoleão de Barros 737, São Paulo, SP 04024002 Brazil; 17 Organização de Procura de Órgãos e Tecidos de Santa Catarina (OPO/SC), Rua Esteves Júnior, 390, Florianópolis, SC 88015130 Brazil; 18 Hospital Sírio-Libanês, R. Dona Adma Jafet, 115, São Paulo, SP Brazil; 19 Universidade Federal do Rio Grande do Sul (UFRGS), Ramiro Barcelos, 2350, Porto Alegre, RS 90035007 Brazil; 20 Faculdade de Medicina de São José do Rio Preto, Av Faria Lima, 5544, São José do Rio Preto, SP 15090000 Brazil; 21 National Institute for Health Technology Assessment, UFRGS, Rua Ramiro Barcelos, 2350, Porto Alegre, RS 90035903 Brazil; 22 Department of Health Research Methods, Evidence, and Impact (HEI), McMaster University, 1280 Main St W, Hamilton, ON Canada

**Keywords:** Guidelines, Organ donation, Intensive care, Brain death, GRADE

## Abstract

**Objective:**

To contribute to updating the recommendations for brain-dead potential organ donor management.

**Method:**

A group of 27 experts, including intensivists, transplant coordinators, transplant surgeons, and epidemiologists, joined a task force formed by the General Coordination Office of the National Transplant System/Brazilian Ministry of Health (CGSNT-MS), the Brazilian Association of Intensive Care Medicine (AMIB), the Brazilian Association of Organ Transplantation (ABTO), and the Brazilian Research in Intensive Care Network (BRICNet). The questions were developed within the scope of the 2011 Brazilian Guidelines for Management of Adult Potential Multiple-Organ Deceased Donors. The topics were divided into mechanical ventilation, hemodynamic support, endocrine-metabolic management, infection, body temperature, blood transfusion, and use of checklists. The outcomes considered for decision-making were cardiac arrest, number of organs recovered or transplanted per donor, and graft function/survival. Rapid systematic reviews were conducted, and the quality of evidence of the recommendations was assessed using the Grading of Recommendations Assessment, Development, and Evaluation (GRADE) system. Two expert panels were held in November 2016 and February 2017 to classify the recommendations. A systematic review update was performed in June 2020, and the recommendations were reviewed through a Delphi process with the panelists between June and July 2020.

**Results:**

A total of 19 recommendations were drawn from the expert panel. Of these, 7 were classified as strong (lung-protective ventilation strategy, vasopressors and combining arginine vasopressin to control blood pressure, antidiuretic hormones to control polyuria, serum potassium and magnesium control, and antibiotic use), 11 as weak (alveolar recruitment maneuvers, low-dose dopamine, low-dose corticosteroids, thyroid hormones, glycemic and serum sodium control, nutritional support, body temperature control or hypothermia, red blood cell transfusion, and goal-directed protocols), and 1 was considered a good clinical practice (volemic expansion).

**Conclusion:**

Despite the agreement among panel members on most recommendations, the grade of recommendation was mostly weak. The observed lack of robust evidence on the topic highlights the importance of the present guideline to improve the management of brain-dead potential organ donors.

## Introduction

Organ donation for transplantation is a complex process led by several health care professionals responsible for a sequence of actions and procedures that begin with identifying a potential organ donor and end with organ procurement surgery and distribution. The progress of this process is essential to increase the deceased-donor pool, and to decrease the growing disparity between the number of patients on transplant waiting lists and the availability of organs [1, 2].

The organ donation process includes the identification of the potential donor, diagnosis of brain death, family support and interview, evaluation of donor eligibility criteria, clinical management of the potential organ donor, and organ procurement and distribution [2, 3]. Given the marked clinical instability that occurs in patients who progress to brain death, the application of potential donor-management strategies aiming at hemodynamic stabilization is crucial to avoid loss of organs due to hypoperfusion or loss of donors due to cardiac arrest. Also, the control of ventilatory support, body temperature, and endocrine-metabolic functions contributes to improving the quality of organs and clinical outcomes in transplant recipients [1, 2, 4, 5].

Despite the lack of evidence on some aspects of the clinical management of potential organ donors, the recommendations presented in this guideline intend to promote a general approach to mitigate the disparity between supply and demand of organs for transplantation.

## Objective

To provide recommendations to guide the clinical management of brain-dead potential organ donors aiming to reduce the rate of cardiac arrest of the potential donor and to improve organ viability for transplantation.

## Method

The present document provides a partial update on the 2011 Brazilian Guidelines for Management of Adult Potential Multiple-Organ Deceased Donors [6–8]. The working group consisted of physicians, nurses, pharmacists, physical therapists, epidemiologists, methodologists, and transplant system managers. The contributions of each participant are shown in Additional file 1, and the respective conflict-of-interest disclosures are shown in Additional file 2.

The target audience of this guideline is health care professionals, especially physicians and nursing staff working in adult ICUs and emergency departments, who are involved in the care of adult individuals with known or suspected brain death.

The clinical issues addressed by the guideline were defined by coordinators of the working group and the methodologists in a face-to-face meeting held in March 2016, after reviewing the recommendations of the 2011 Brazilian Guidelines for Management of Adult Potential Multiple-Organ Deceased Donors [6–8]. The issues were prioritized according to the perception of their impact on medical management and variability in clinical practice and divided into the following major topics: (1) ventilatory support; (2) hemodynamic support; (3) endocrine, metabolic and nutritional management; (4) specific aspects that include infection and sepsis, red blood cell transfusion, and body temperature control; and (5) goal-directed therapy. For each clinical issue, operational questions were developed and framed using the PICO (population-intervention-comparison-outcome) format. The population of interest consists of potential organ donors with known or suspected brain death [3], hereafter referred to as potential donors. The outcomes considered for decision-making were cardiac arrest, the number of organs recovered or transplanted per donor, and graft function or graft survival.

For each clinical issue, rapid systematic reviews [9, 10] were conducted using the following search strategy: (1) Review of the reference lists of Brazilian guidelines [6–8] and the Society of Critical Care Medicine (SCCM) [11] statement on the management of the potential organ donor; (2) Review of related topics in the DynaMed and UpToDate databases; and (3) PubMed search focusing on systematic reviews and clinical trials published until October 2016 and until January 2017. Quality of evidence was assessed using the Grading of Recommendations Assessment, Development, and Evaluation (GRADE) system [12].

The recommendations were prepared and submitted to two face-to-face expert panels held in November 2016, and February 2017. For each recommendation, the direction of the course of action was discussed (whether to perform or not to perform the proposed action), and the strength of the recommendation was classified as strong or weak according to the GRADE system [12]. After the last panel meeting, a new systematic search covering the period from October 2016 to May 2020 was carried out to identify new evidence that could potentially modify the recommendations. From June to July 2020, a Delphi process was performed with the panelists to present the results of the literature update and review the direction and strength of the recommendations.

## Results

**Fig. 1:**
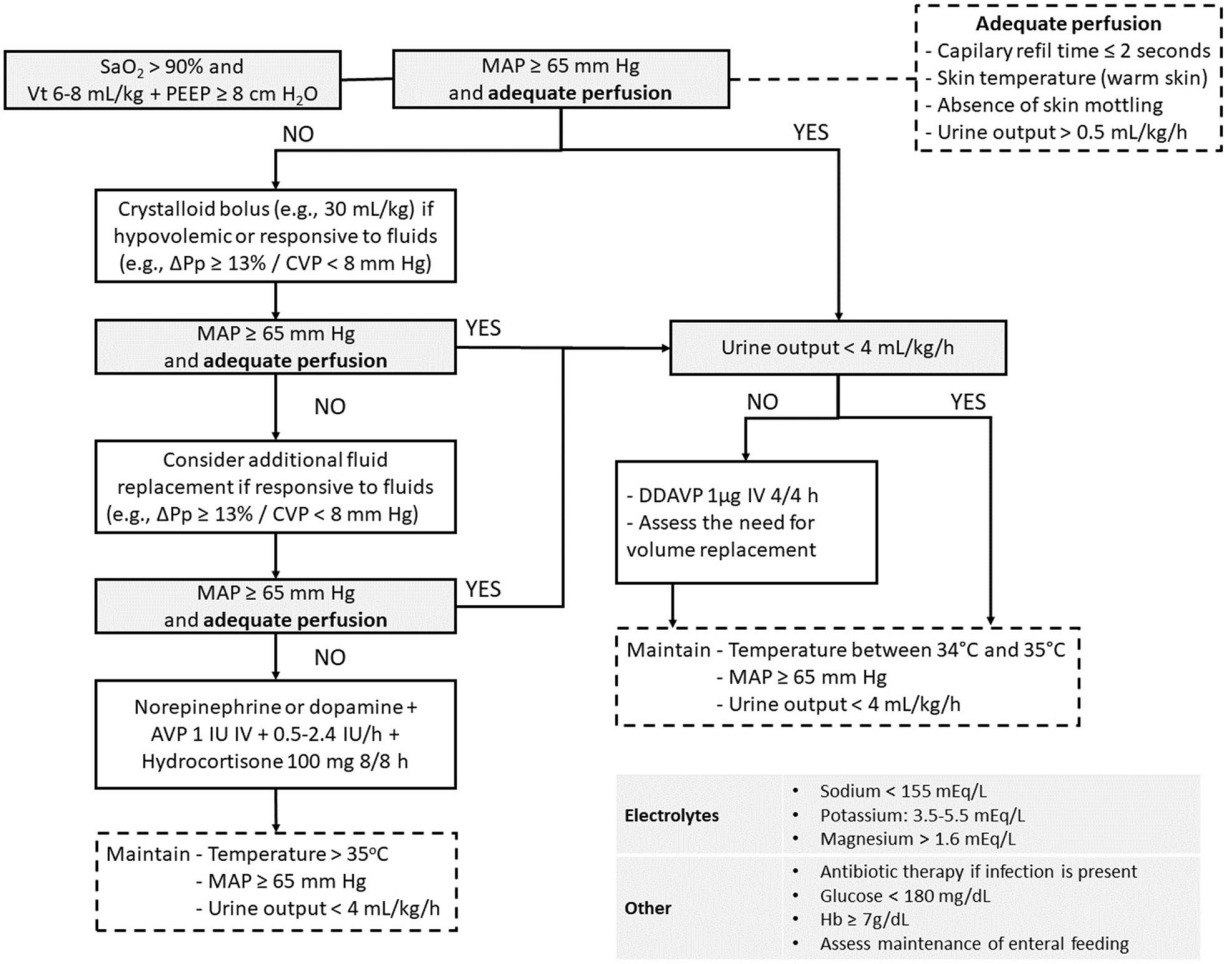
Flow of the recommendations

A total of 19 recommendations were drawn from the expert panel. Of these, 7 were classified as strong, 11 as weak, and 1 was considered as good clinical practice. Table 1 shows a summary of the recommendations. Figure 1 presents graphically the flow of the recommendations along the clinical management. Additional file 3 provides a checklist with the main recommendations with a positive direction of action to assist in bedside monitoring of clinical goals related to the recommendations and in the application of the management strategies.

**Table 1:** Summary of recommendations

Recommendations	Level of evidence	Grade of recommendation	Practical considerations
Ventilatory support			
1. We recommend using a lung-protective ventilation strategy in all PDs	Low	Strong	Vt between 6 and 8 mL/kg of predicted body weight and PEEP of 8–10-cm H_2_O Adjust FiO_2_ and PEEP to obtain SaO_2_ > 90% Perform apnea testing with CPAP
2. We suggest not using ARM routinely in PDs	Very low	Weak	ARM can be considered if there is refractory hypoxemia in hemodynamically stable PDs
Hemodynamic support			
3. We recommend performing initial volemic expansion in hemodynamically unstable PDs with hypovolemia or responsive to fluids according to fluid responsiveness assessment		Good clinical practice	Initial volume expansion with 30 mL/kg of crystalloids Assess fluid status and responsiveness for additional fluid replacement Preferably use dynamic parameters Neutral or negative fluid balance after achieving hemodynamic stability
4. We recommend administering norepinephrine or dopamine to control blood pressure in PDs who remain hypotensive after volemic expansion	Very low	Strong	Start adrenergic vasopressors to obtain a MAP ≥ 65 mm Hg Dopamine is the vasopressor of choice when there is bradycardia Consider the potential arrhythmogenic effect of dopamine, which implies the risk of PD loss due to cardiac arrest
5. We suggest not using low-dose dopamine for renal protection in PDs	Very low	Weak	Consider the potential arrhythmogenic effect of dopamine, which implies the risk of PD loss due to cardiac arrest
Endocrine and electrolyte management			
6. We recommend combining AVP in PDs receiving norepinephrine or dopamine	Low	Strong	Combine AVP (1 IU bolus + 0.5–2.4 IU/h) with norepinephrine or dopamine
7. We recommend administering AVP or DDAVP to control polyuria in PDs with diabetes insipidus	Low	Strong	AVP if vasopressors are required. DDAVP (1–2-µg IV 2–4 h) if vasopressors are not required
8. We suggest combining low-dose corticosteroids in PDs receiving norepinephrine or dopamine	Low	Weak	Combine 300 mg IV/day in PDs with norepinephrine or dopamine
9. We suggest not using thyroid hormones routinely in PDs	Very low	Weak	There are no hemodynamic benefits They can be considered if prolonged management is required
10. We suggest performing glycemic control in PDs	Very low	Weak	Administer insulin to achieve a glucose level of 140–180 mg/dL Monitor blood glucose at least every 6 h
11. We suggest maintaining serum sodium levels < 155 mEq/dL in PDs	Very low	Weak	Correct water deficit with hypotonic fluids Correct hypovolemia
12. We recommend maintaining serum potassium levels between 3.5 and 5.5 mEq/L in PDs	Very low	Strong	
13. We recommend maintaining serum magnesium levels > 1.6 mEq/L in PDs	Very low	Strong	
Other aspects			
14. We suggest maintaining nutritional support in PDs if well tolerated	Very low	Weak	
15. We recommend using antibiotics in PDs with infection or sepsis	Low	Strong	Maintain appropriate antibiotic therapy in the donor for at least 24 h Collect cultures from different sites in all donors
16. We suggest maintaining body temperature above 35 °C in hemodynamically unstable PDs	Very low	Weak	Monitor core temperature Prevent and treat hypothermia in PDs receiving vasoactive amines
17. We suggest inducing hypothermia (34–35 °C) in PDs without hemodynamic instability	Low	Weak	Monitor core temperature Induce hypothermia by applying ice packs in PDs not receiving vasoactive amines
18. We suggest transfusing packed red blood cells in PDs with hemoglobin levels < 7 g/dL	Very low	Weak	
19. We suggest using goal-directed protocols during the management of PDs	Very low	Weak	Monitor care using evidence-based clinical goal-directed checklists

PD: potential donor; Vt: total volume; PEEP: positive-end expiratory pressure; SaO_2_: arterial oxygen saturation; CPAP: continuous positive airway pressure; ARM: alveolar recruitment maneuver; MAP: mean arterial pressure; AVP: arginine-vasopressin; DDAVP: 1-deamino-8-d-arginine-vasopressin; IV: intravenous

### Ventilatory support

1. We recommend using a lung-protective ventilation strategy in all potential donors (low level of evidence, strong recommendation).

*Summary of evidence* In potential donors, an initially normal or near-normal lung function (PaO_2_/FiO_2_ ≥ 300) may deteriorate due to common complications in critical patients, such as pulmonary contusion, lung injury following blood transfusion, pneumonia, atelectasis, and mechanical ventilation-related iatrogenic injuries [13–18]. In addition, approximately 30–45% of potential donors develop acute respiratory distress syndrome (ARDS; PaO_2_/FiO_2_ < 300), and only 15–20% of the lungs are suitable for transplantation at the end of the procurement process [13, 15, 17]. The lung-protective ventilation strategy in potential donors with normal lungs and the apnea testing performed with continuous positive airway pressure (CPAP) have been associated with an increase in eligibility for lung donation [18–20].

*Remarks* The protective ventilation strategy for healthy lungs consists of the combination of a tidal volume of 6–8 mL/kg and PEEP of 8–10-cm H_2_O. To promote adequate blood oxygenation, FiO_2_ and PEEP must be adjusted to obtain a SaO_2_ > 90%. To avoid atelectasis, the apnea test with 10 cm H_2_O CPAP can be performed using a closed-circuit system in potential donors with preserved lungs who are candidates for lung procurement, or even when hypoxemic respiratory failure is present. Also, the same procedure can be considered on those who have failed the test due to hypoxemia after disconnection.

2. We suggest not using alveolar recruitment maneuvers routinely in potential donors (very low level of evidence, weak recommendation).

*Summary of evidence* Although alveolar recruitment maneuvers have been suggested for the ventilatory management of organ donors with lung injury (PaO_2_/FiO_2_ < 300) [13–16, 18, 20], and these maneuvers could reduce hypoxemia after apnea testing, contributing to increasing the viability of pulmonary grafts [14–18, 20], a randomized clinical trial showed unfavorable outcomes in critically ill patients [21]. Besides, no randomized studies have demonstrated their efficacy in the population of potential donors.

*Remarks* Performing alveolar recruitment maneuvers in hemodynamically stable potential donors is probably feasible in units with experience in the management of ARDS. In cases of hypoxemia refractory to the lung-protective ventilation strategy, however, alveolar recruitment maneuvers should not be performed routinely. Their use is not indicated in hemodynamically unstable potential donors.

### Hemodynamic support

#### Volemic expansion and vasopressors

3. We recommend performing initial volemic expansion in hemodynamically unstable potential donors with hypovolemia or responsive to fluids according to fluid responsiveness assessment (good clinical practice).
4. We recommend administering norepinephrine or dopamine to control blood pressure in potential donors who remain hypotensive after volemic expansion (very low level of evidence, strong recommendation).

*Summary of evidence* Potential donor hypotension is associated with a higher incidence of postoperative liver graft dysfunction and longer hospital stay in liver transplant recipients [22, 23]. Targeting a mean arterial pressure (MAP) ≥ 65 mm Hg has also been associated with reduced occurrence of cardiac arrest in potential donors [22, 24]. Intravascular volume expansion guided by ventricular filling pressures or respiratory pulse pressure variation (PPV) in hemodynamically unstable potential donors is associated with faster recovery of renal graft function and reduced circulating levels of inflammatory cytokines [22, 25]. A randomized trial detected no difference between usual fluid management or fluid management directed by a PPV and cardiac index. On the other hand, there was a trend toward an increase in the number of organs transplanted per donor among unstable potential donors responsive to fluids (*p* = 0.059) [26].

Conversely, avoiding fluid overload after the initial volume resuscitation to stabilize blood pressure seems to be beneficial. This approach is associated with a greater number of organs transplanted per donor and a greater number of lungs transplanted without reducing the number of other donated organs or impairing survival in the heart, liver, pancreas, or kidney transplant recipients [19, 27–29].

If hypotension persists after adequate volume resuscitation, adrenergic vasopressors should be used to achieve adequate blood-pressure levels [30]. There is no difference in clinical outcomes in studies comparing norepinephrine and dopamine [31–33]. Disruption of vagal activity secondary to brain death may result in atropine-refractory bradycardia. In these cases, adrenergic drugs as isoproterenol, epinephrine, and dopamine have been suggested as positive chronotropic agents to treat bradycardia in potential donors. Considering the predominance of noradrenaline action on alpha-1 receptors, its infusion usually occurs without significant increase in heart rate. Hence, dopamine or epinephrine may be more convenient for the treatment of hypotension due to a positive chronotropic effect [6, 34, 35].

*Remarks* Obtaining an MAP ≥ 65 mm Hg as a blood-pressure target contributes to the perfusion of organs that are intended to be preserved for transplantation [22–24]. Hypovolemia is very frequent in potential organ donors and should be considered when hypotension is present. The initial infusion of crystalloids (e.g., 30 mL/kg) in potential donors who are hypovolemic or responsive to fluids (when any fluid responsiveness assessment parameter is already available) contributes to blood-pressure control by improving tissue perfusion [24–26].

Conversely, fluid overload should be avoided [19, 27–29]. Assessment of fluid responsiveness with static variables (e.g., central venous pressure—CVP) and/or dynamic parameters (e.g., PPV) can be used to guide volume replacement, helping to prevent fluid overload. Dynamic parameters can more accurately discriminate between responsive and unresponsive individuals [30–38]. Once hemodynamic stability is achieved, strategies aimed at neutral fluid balance may be more beneficial [19, 27–29].

If the blood-pressure target is not achieved with the initial volume expansion, norepinephrine or dopamine infusion should be started immediately. The use of dopamine is likely advantageous for cases of bradycardia with signs of low cardiac output [6, 34, 35], but the arrhythmogenic potential of dopamine should be considered [39].

5. We suggest not using low-dose dopamine for renal protection in potential donors (very low level of evidence, weak recommendation).

*Summary of evidence* A cohort study of 93 heart transplant recipients showed that pretreatment with low-dose dopamine (4 μg/kg/min) in heart donors was associated with higher graft survival 3 years after transplantation (87.0 vs. 67.8%, *p* < 0.03) [40]. A randomized-controlled trial of 264 organ donors reported that the administration of low-dose dopamine reduced the need for hemodialysis in recipients (OR 0.54; 95% CI 0.35–0.83), but with no benefits for kidney graft survival after 3 years [41]. In the 5-year follow-up analysis of 487 renal transplant recipients from the same trial, the researchers failed to show a significant advantage of dopamine administration in potential donors to long-term kidney graft survival, although time of dopamine infusion and graft failure were exposure-related (HR 0.96; 95% CI 0.92–1.00, per hour) [42]. The same group reported that low-dose dopamine did not negatively affect the short- or long-term outcomes after liver transplants [43].

*Remarks* Although the administration of low-dose dopamine in potential donors reduces the need for multiple dialysis sessions, the long-term benefits for heart and kidney graft survival are unclear. The panel considered the potential arrhythmogenic effect of dopamine, which may imply a greater risk of loss of potential donors due to cardiac arrest before organ procurement.

### Endocrine and electrolyte management

#### Hormones

6. We recommend combining arginine vasopressin (AVP) in potential donors receiving norepinephrine or dopamine to control blood pressure (low level of evidence, strong recommendation).

*Summary of evidence* The use of AVP in brain-dead potential donors contributes to reducing the need for adrenergic vasopressors and is associated with a lower incidence of cardiovascular deterioration and cardiac arrest [44–48], in addition to contributing to the control of plasma hyperosmolarity [46]. AVP infusion allows, in some cases, complete discontinuation of adrenergic vasopressors without causing adverse effects on the function of organs transplanted [48, 49]. Finally, AVP infusion seems to be associated with a greater number of donated organs and a lower rate of graft refusal due to organ dysfunction [45].

*Remarks* The administration of an initial 1 IU AVP bolus followed by infusion of 0.5 IU/h to 2.4 UI/h helps to maintain blood pressure in potential donors requiring vasopressors, and contributes to the control of polyuria and normovolemia in the presence of diabetes insipidus [44–46, 48, 49]. AVP should be started at the same time of adrenergic vasopressor infusion.

7. We recommend administering AVP or 1-deamino-8-d-arginine vasopressin (DDAVP) to control polyuria in potential donors with diabetes insipidus (low level of evidence, strong recommendation).

*Summary of evidence* The analysis of the database of a randomized clinical trial that evaluated 487 renal graft recipients showed better control of daily urine output (*p* < 0.001) and a lower need for fluids in the DDAVP group (*p* < 0.001). DDAVP was associated with improved renal graft survival (85.4% vs. 73.6%, *p* = 0.003) after 2 years, with no differences in acute rejections (OR 1.32; 95% CI 0.70–2.49) or delayed graft function (OR 0.97; 95% CI 0.57–1.65) [50].

*Remarks* DDAVP acts exclusively on V2 receptors and is indicated to control polyuria (urine output > 4 mL/kg/h) in potential donors with diabetes insipidus who maintain adequate blood pressure without adrenergic vasopressors. AVP is preferred to control polyuria in potential donors with diabetes insipidus who need adrenergic vasopressors. The combination of AVP and DDAVP may be considered in refractory cases [51]. Although the intranasal route is feasible, DDAVP should preferably be administered intravenously, at a dose of 1–2 µg every 2–4 h [8, 13, 15], until a urine output < 4 mL/kg/h has been achieved [50–53].

8. We suggest using low-dose corticosteroids in potential donors receiving norepinephrine or dopamine to control blood pressure (low level of evidence, weak recommendation).

*Summary of evidence* A small retrospective study reported that administration of 15-mg/kg methylprednisolone was associated with higher PaO_2_/FiO_2_ values (*p* = 0.01) and a greater number of lungs transplanted (*p* < 0.01) [54]. Conversely, a before-and-after study comparing 15-mg/kg methylprednisolone with 300-mg hydrocortisone found no difference in the oxygenation and hemodynamic stability of the potential donor or in the number of organs transplanted [55]. A recent small randomized-controlled trial showed that a single dose of 15 mg/kg/day of methylprednisolone administered to the potential organ donor may negatively affect the graft function by increasing the antigenicity of the kidneys before transplantation. This negative effect was not noticed among brain-dead donors who received 15 mg/kg/day of methylprednisolone followed by 100 mg every 2 h until organ harvesting [56]. Eleven randomized-controlled trials analyzed in a systematic review did not support the use of high-dose corticosteroids in the management of potential donors [57]. On the other hand, a randomized multicenter cluster study including 259 individuals compared the administration of low-dose hydrocortisone (300 mg/day) with no corticosteroids. The doses (*p* = 0.03) and duration of infusion (*p* < 0.001) of vasopressors were lower in the intervention group, and the complete vasopressor withdrawal was 4.7 times more frequent in the corticosteroid group [58].

*Remarks* Despite conflicting evidence, the use of corticosteroids is of low cost and a low risk to potential donors and may have a positive effect on hemodynamic outcomes; therefore, their use is indicated in these patients. Current evidence does not suggest ventilatory or hemodynamic benefits associated with corticosteroid therapy at high doses compared with low doses (i.e., 100 mg every 8 h). Higher doses should be avoided.

9. We suggest not using thyroid hormones routinely in potential donors (very low level of evidence, weak recommendation).

*Summary of evidence* Administration of thyroid hormones in potential donors did not add any benefit, such as a reduction in vasopressor use, an increase in cardiac index, or an increase in organ procurement for transplantation [59–65]. Observational studies had suggested an increase in heart procurement, which was not confirmed in randomized clinical trials [66, 67], even in brain-dead organ donors with hemodynamic instability and/or impaired cardiac function [68, 69].

*Remarks* Brain death is associated with a drop in circulating thyroid hormone levels, which could contribute to hemodynamic instability; however, there is no evidence to support the use of thyroid hormones in potential donors, given their costs and risks.

10. We suggest performing glycemic control in potential donors (very low level of evidence, weak recommendation).

*Summary of evidence* Four observational studies evaluated the effect of potential donor hyperglycemia on post-transplant pancreatic function [70–73]. One study showed a correlation between donor blood glucose immediately before organ retrieval and HbA1C 1 year after transplantation [73], and another study found an association between hyperglycemia and graft loss (HR 1.4; *p* = 0.03) [74]. Two studies showed no association between potential donor blood glucose and post-transplant pancreatic graft function [70–72]. One observational study found an association between glycemic control and creatinine of the potential donor before organ retrieval [75]. Conversely, there is no evidence that hyperglycemia is associated with liver graft dysfunction [76]. A study of 1611 potential donors reported that a glucose level < 180 mg/dL was an independent predictor of four or more organs transplanted per donor (OR 1.35; 95% CI 1.01–1.82) [77]. A set of potential donor care measures, including glycemic control, was associated with achieving ≥ 3 organs transplanted per donor (OR 1.9; 95% CI 1.35–2.68), but it was not possible to assess the isolated effect of glycemic control [78].

*Remarks* Very-low-quality evidence suggests that a glucose level < 180 mg/dL is associated with a greater number of organs transplanted. Blood glucose should be monitored in all potential donors at least every 6 h, targeting levels of 140–180 mg/dL, and intravenous insulin infusion can be used to this end.

#### Electrolytes

11. We suggest maintaining serum sodium levels below 155 mEq/dL in potential donors (very low level of evidence, weak recommendation).

*Summary of evidence.* Five descriptive observational studies were identified (*n* = 5733), which evaluated only graft viability/function. In four of these studies (*n* = 5545), there was no negative effect of donor hypernatremia above 155 mEq/L on liver or heart graft function [79–82]. In only one study (*n* = 188), hypernatremia was associated with more cases of early graft loss [83]. Some authors have suggested that deceased-donor hypernatremia may be a factor for worse prognosis of graft function, but these findings have not been universally confirmed [79–85]. Changes in natremia may reflect inadequate volume management, especially in the presence of diabetes insipidus, one of the reasons for its correction [11].

*Remarks* Hypernatremia is often associated with hypovolemia, and should be controlled with volume expansion, replacement of hypotonic fluids, and control of polyuria with AVP or DDAVP. Serum sodium should be monitored, targeting levels < 155 mg/dL.

12. We recommend maintaining serum potassium levels between 3.5 and 5.5 mEq/L in potential donors (very low level of evidence, strong recommendation).

*Summary of evidence* There are no studies that directly evaluate the effect of hyper- or hypokalemia in potential donors. A comparison of potassium levels in ICU patients showed that hyperkalemia was more common in patients who died (9.2% vs. 0.9%, *p* < 0.001) and that serum potassium concentration could be a predictor of death in critically ill patients [86].

*Remarks* Despite the absence of studies directly evaluating the effects of potential donor serum potassium levels, potassium is a determining factor in the resting potential of electrically sensitive cells. Changes in potassium levels are related to cardiac arrhythmias and may compromise the management of potential donors. Potassium levels should be monitored, and usual correction measures should be implemented, targeting serum levels between 3.5 and 5.5 mEq/L.

13. We recommend maintaining serum magnesium levels above 1.6 mEq/L in potential donors (very low level of evidence, strong recommendation).

*Summary of evidence* Studies on the influence of serum magnesium levels were found in critically ill patients, but none in potential donors [87–92]. Two observational studies and one randomized study identified an association between hypomagnesemia and higher mortality in critically ill patients [87, 88, 91], in addition to a greater likelihood of QT interval prolongation (OR 42.8; 95% CI 14.5–126.2) [88]. This association of hypomagnesemia with mortality was reinforced in a systematic review [89]. In addition to being arrhythmogenic, hypomagnesemia appears to be associated with non-recovery of renal function in patients with acute kidney injury (70% vs. 31%, *p* = 0.003) [92].

*Remarks* Hypomagnesemia is associated with cardiac arrhythmias and worse prognosis in critically ill patients, with no direct evidence in brain-dead potential donors. However, this is a low-cost procedure, and in the ICU setting, routine monitoring until normalization of magnesium levels is a common practice, which may be beneficial for potential donors. Magnesium levels should be monitored, and magnesium sulfate should be administered, as usual, targeting serum levels above 1.6 mEq/L.

### Other aspects of potential donor management

#### Nutritional support

14. We suggest maintaining nutritional support in potential donors if well tolerated (very low level of evidence, weak recommendation).

*Summary of evidence* Although there is no evidence on nutritional support, different guidelines recommend continuing nutritional support of the donor in the absence of contraindications [7, 9, 51]. Possible benefits include increased liver glycogen reserves, which could positively influence the liver graft [93, 94], and maintenance of intestinal mucosal trophism, which could reduce the potential for bacterial translocation.

*Remarks* For brain-dead individuals requiring ICU management for prolonged periods (e.g., brain-dead pregnant women; prolongation of the diagnostic process or the family decision for donation), it is reasonable that energy expenditure should be estimated or measured [95], considering that baseline energy expenditure is 15–30% lower in brain-dead individuals than in other critically ill patients [96]. Thus, in individuals already receiving full nutritional support, energy intake may be reduced once brain death is established. A minimum energy intake (e.g., 500 kcal) could be considered in potential donors who had not been on enteral feeding before brain death was diagnosed, taking into account its potential benefit in the maintenance of intestinal mucosal trophism. However, it does not seem appropriate to start enteral feeding when the organs are likely to be harvested within a short period or in the presence of any of the usual contraindications to initiate/maintain enteral feeding (e.g., gastrointestinal tract obstruction, ileus, vomiting/aspiration of gastric contents, severe hemodynamic instability, and high doses of vasopressors).

#### Infection and sepsis

15. We recommend using antibiotics in potential donors with infection or sepsis (low level of evidence, strong recommendation).

*Summary of evidence* Different observational studies evaluated the transmission of bacterial infection in organ donors with culture-proven infection. The most commonly observed microorganisms were Staphylococcus aureus, *Streptococcus* sp*., Klebsiella* sp., and *Acinetobacter baumannii*. Bacterial transmission is rarely observed [97–104], provided that donors with evidence of infection receive appropriate antibiotic therapy [97–102, 105, 106]. The duration of donor antibiotic therapy ranged from 24 to 96 h in different studies [97, 99, 102, 105]. Also, different authors have reported maintaining the same antibiotics administered to the donors in the transplant recipients, for periods ranging from 7 to 14 days [98, 100, 105, 106]. The presence of donor infection had no impact on the survival of grafts or transplant recipients [97–102, 105, 106].

*Remarks* The risk of transmission of bacterial infection from organ donors to recipients is low, and donor infection does not appear to negatively affect outcomes. The risks are lower with appropriate antibiotic therapy in the donor for at least 24 h, followed by maintenance of the antibiotic in the recipient for 7–14 days [97–102, 105, 106]. Some donors have subclinical bacteremia at the time of organ procurement; therefore, cultures should be collected from blood and different sites in all donors, and the recipient antibiotic therapy should be directed by the results of culture [99, 107–110].

#### Body temperature control

16. We suggest maintaining body temperature above 35 °C in hemodynamically unstable potential donors (very low level of evidence, weak recommendation).
17. We suggest inducing moderate hypothermia (34–35 °C) in potential donors without hemodynamic instability (low level of evidence, weak recommendation).

*Summary of evidence* Delayed renal graft function was evaluated in a randomized-controlled trial that compared hypothermia (34–35 °C) versus usual management (36.5–37.5 °C) in 370 potential donors without hemodynamic instability. The main result was a reduction in delayed renal graft function among recipients (OR 0.62; 95% CI 0.43–0.92). There was no difference in the number of organs transplanted per donor, adverse events, or cardiac arrest [111]. Two retrospective cohort studies nested in the randomized dopamine trial demonstrated that spontaneous donor hypothermia was associated with lower creatinine levels before organ procurement without effect on kidney graft survival [112], and with an unfavorable clinical course after heart transplant [113]. In a clinical population of post-cardiac arrest patients, i.e., patients at increased risk of hemodynamic instability, a meta-analysis of five clinical trials found a higher risk of recurrent arrest in patients with induced hypothermia (< 35 °C) in prehospital management (RR 1.23; 95% CI 1.02–1.48) [114].

*Remarks* Hypothermia is a low-cost intervention [115] associated with better renal graft function, but it can increase the risk of cardiac arrest in the potential donor [111, 114]. The risk appears to be low in hemodynamically stable potential donors, in whom the use of hypothermia can be justified by improved graft viability. In the presence of hemodynamic instability [111], normothermia (> 35 °C) should be maintained in potential donors to reduce the risk of cardiac arrest [114]. Induction of moderate hypothermia (34–35 °C) is considered a simple (application of ice packs) and inexpensive approach, but it is important to monitor core temperature, which is not available in all ICUs.

#### Red blood cell transfusion

18. We suggest transfusing packed red blood cells in potential donors with hemoglobin levels < 7 g/dL (very low level of evidence, weak recommendation).

*Summary of evidence* The systematic literature search identified 1 descriptive observational study that evaluated function in 1884 renal grafts from 1006 brain-dead donors. Among donors, 52% received blood transfusion. Renal grafts from transfused donors had a lower rate of delayed graft function than those from non-transfused donors (26% vs. 34%, *p* < 0.001). The criteria defining the need for blood transfusion were not identified [116].

*Remarks* Anemia can compromise oxygen transport and delivery to the organs that are intended to be preserved for transplantation. However, we are unaware of the hemoglobin concentration necessary to contribute to adequate oxygen transport and delivery in potential donors. Considering the high cost and frequent shortage of blood products for transfusion, the decision to transfuse should not differ from the usual practice in other critically ill patients.

#### Goal-directed protocols

19. We suggest using a goal-directed protocol during the management of potential donors (very low level of evidence, weak recommendation).

*Summary of evidence* Although there is no consistent evidence about an individual treatment that will improve the number and quality of donated organs [117], observational studies have reported that combining different treatments through the application of a potential donor-management protocol is associated with a higher organ yield for transplantation [24, 78, 118–124], lower incidence of delayed renal graft function [111], greater eligibility for lung donation [19, 28], and lower incidence of donor losses due to cardiac arrest [19, 24, 28, 119, 120]. In general, the outcomes are associated with the number of goals achieved during potential donor management, including ventilatory, hemodynamic, and endocrine-metabolic management goals [24, 78, 121–123]. In seven studies, the use of a checklist helped implement the goal-directed protocols and may have positively influenced the results [19, 28, 78, 121, 124–126].

*Remarks* The application of a potential donor-management protocol guided by a clinical goal-directed checklist may contribute to increasing the number of organs transplanted per donor, influence graft function, and reduce donor losses due to cardiac arrest.

## General considerations and future directions

The present guideline aimed to provide parameters to optimize the clinical management of potential donors based on the available evidence, aiming to improve the quality of organs for transplantation and to reduce donor losses. However, it is well known that it may take years for a large-scale translation of the best scientific evidence into effective practice. Thus, establishing clinical protocols can help to reduce the time required to incorporate best practices. The use of a goal-directed checklist can play an important role in the adjustment of approaches and adherence to the best evidence in complex procedures [127–130].

This guideline evaluated a broad volume of treatments and we performed rigorous PICO-driven research to provide the recommendations based on standardized rapid review methods [9, 10]. Potential limitations are the low or very low certainty in the evidence identified for many of the questions, and indirect evidence that did not change after the systematic review update. However, management recommendations are consistent with similar documents recently published [11, 131, 132].

Several challenges regarding ethical, infrastructure, and operational issues are faced while planning and conducting studies that involve potential organ donors, which results in few randomized clinical trials [133]. The scarcity of studies with such methodological strength implies uncertainties about some interventions such as low-dose dopamine and moderate hypothermia, which, despite appearing to be related to renal graft benefit, may result in cardiac arrhythmias and hemodynamic instability. In this context, developing clinical trials in this field of medical knowledge may be helpful to understand some important aspects in the management of the potential organ donor.

Some observational studies have reported that the application of a checklist to guide the management of brain-dead potential donors may help to reduce the rate of cardiac arrest in potential donors and increase the number of organs recovered per donor [24, 78, 119, 121, 122, 124, 126, 134, 135]. In this context, we used the main recommendations of the present guideline to develop an evidence-based clinical goal-directed checklist (Additional file 3) with the purpose of providing transplant coordinators and ICU professionals with essential information to optimize the care of potential donors.

However, because the available studies highlighting the role of potential donor-management checklists are observational, there is insufficient evidence to support the systematic use of checklists in the management of potential donors. Therefore, we proposed the Donation Network to Optimize Organ Recovery Study (DONORS; NCT03179020), which is a parallel cluster randomized-controlled multicenter trial that aims to test the effectiveness of the implementation of a checklist containing goals and recommendations of care in reducing organ donor losses due to cardiac arrest and increasing the number of organs recovered per donor [136].

The implementation of the checklist should be preceded by the appropriate training of intensive care teams and transplant coordinators. We suggest applying the checklist at the bedside immediately after the first clinical examination for the diagnosis of brain death, repeating the application, ideally, every 6 h until organ procurement for transplantation. We also suggest that a member of the transplant coordination office or a designated professional of the ICU or emergency department applies the checklist at the bedside. The same individual will also be responsible for personally prompting the physician in charge to modify the clinical management if any inappropriate aspect of care, according to the checklist, is noted.

## Supplementary information

**Additional file 1.** Working group and contributions of each participant.

**Additional file 2.** Declaration of competing interests.

**Additional file 3.** Checklist for clinical management of brain-dead potential organ donor.

## Data Availability

All relevant data are within the paper and its additional files.
