# Supplementary material for: Brazilian guidelines for the management of brain-dead potential organ donors. The task force of the AMIB, ABTO, BRICNet, and the General Coordination of the National Transplant System
Source: Ann Intensive Care. 2020 Dec 14;10:169. doi: 10.1186/s13613-020-00787-0 (PMC7736434; doi:10.1186/s13613-020-00787-0)
Supplement: Supplementary file 1 — Additional file 1. Working group and contributions of each participant. [file 13613_2020_787_MOESM1_ESM.docx]

**Additional file 1**. Working group and the contributions of each participant.

| **Name** | **Contribution** | **Attended the scope meeting (January 26, 2016)** | **Searched the literature and reviewed evidence** | **Attended the 1st recommendations meeting (November 3, 2016)** | **Attended the 2nd recommendations meeting (February 9, 2017)** | **Answered Delphi**  **(June, 15 to July, 10, 2020)** | **Reviewed and approved the final document** |
| --- | --- | --- | --- | --- | --- | --- | --- |
| Alexandre Biasi Cavalcanti. Intensivist. | Panel member |  | x | x | x | x | x |
| Anderson Ricardo Roman Gonçalves. Nephrologist. | Technical review |  |  |  |  | x | x |
| Caroline Cabral Robinson. Physical Therapist. | Methodologist | x |  | x | x |  | x |
| Cátia Moreira Guterres. Pharmacist. | Methodologist | x |  | x | x |  | x |
| Cassiano Teixeira. Intensivist. | Panel member | x | x | x | x | x | x |
| Cinara Stein. Physical Therapist. | Methodologist |  | x | x | x |  | x |
| Cristiano Augusto Franke. Intensivist. | Panel member |  |  | x | x |  | x |
| Daiana Barbosa da Silva. Nurse. | Panel member |  |  | x | x |  | x |
| Daniela Ferreira Salomão Pontes. Intensivist. | Technical review |  |  |  |  | x | x |
| Diego Silva Leite Nunes. Intensivist. | Panel member |  |  | x |  | x | x |
| Edson Abdala. Intensivist. | Panel member |  | x | x |  | x | x |
| Felipe Dal Pizoll. Intensivist. | Panel member |  | x |  |  | x | x |
| Fernando Augusto Bozza. Intensivist. | Panel member |  |  |  | x | x | x |
| Flávia Ribeiro Machado. Intensivist. | Panel member |  | x | x | x | x | x |
| Glauco Adrieno Westphal. Intensivist. | Panel member Co-chair of the guideline. | x | x | x | x | x | x |
| Joel de Andrade. Intensivist. | Panel member |  |  | x |  | x | x |
| Luciane Nascimento Cruz. Psychiatrist. | Methodologist |  |  |  | x |  | x |
| Luciano César Pontes de Azevedo. Intensivist. | Panel member |  | x | x | x | x | x |
| Maicon Falavigna. Internist. | Methodologist. Co-chair of the guideline. | x | x | x | x | x | x |
| Miriam Cristine Vahl Machado. Intensivist. | Panel member |  | x | x |  | x | x |
| Regis Goulart Rosa. Intensivist. | Panel member |  |  | x |  | x | x |
| Roberto Ceratti Manfro. Nephrologist. | Technical review |  |  |  |  | x | x |
| Rosana Reis Nothen. Pediatrician. | Technical review |  |  |  |  |  | x |
| Suzana Margareth Lobo. Intensivist. | Panel member |  | x | x |  | x | x |
| Tatiana Helena Rech. Intensivist. | Panel member |  | x | x |  | x | x |
| Thiago Lisboa. Intensivist. | Panel member |  |  | x | x | x | x |
| Verônica Colpani. Physical Therapist. | Methodologist |  | x | x | x |  | x |
