# Supplementary material for: Brazilian guidelines for the management of brain-dead potential organ donors. The task force of the AMIB, ABTO, BRICNet, and the General Coordination of the National Transplant System
Source: Ann Intensive Care. 2020 Dec 14;10:169. doi: 10.1186/s13613-020-00787-0 (PMC7736434; doi:10.1186/s13613-020-00787-0)
Supplement: Supplementary file 2 — Additional file 2. Declaration of competing interests. [file 13613_2020_787_MOESM2_ESM.docx]

**Additional file 2**. Declaration of interests.

Summary table of conflicts of interest declared by the guideline participants

| Name | Conflict of interest declared | | | Decision on participation |
| --- | --- | --- | --- | --- |
|  | Question | Description of conflict of interest | |  |
| Alexandre Biasi Cavalcanti | none | | | Not applicable |
| Anderson Ricardo Roman Gonçalves | none | | | Not applicable |
| Caroline Cabral Robinson | none | | | Not applicable |
| Cátia Moreira Guterres | none | | | Not applicable |
| Cassiano Teixeira | none | | | Not applicable |
| Cinara Stein | none | | | Not applicable |
| Cristiano Augusto Franke | none | | | Not applicable |
| Daiana Barbosa da Silva | none | | | Not applicable |
| Daniela Ferreira Salomão Pontes | 5b | Coordinator of the National Transplant System of the Brazilian Ministry of Health | | Did not participate in the panel |
| Diego Silva Leite Nunes | 5b | Health regulation consultant of the General Coordination Office of the National Transplant System of the Brazilian Ministry of Health | | Declared and participated |
| Edson Abdala | none | | | Not applicable |
| Felipe Dal-Pizoll | none | | | Not applicable |
| Fernando Augusto Bozza | none | | | Not applicable |
| Flávia Ribeiro Machado | none | | | Not applicable |
| Glauco AdrienoWestphal | none | | | Not applicable |
| Joel de Andrade | none | | | Not applicable |
| Luciane Nascimento Cruz | none | | | Not applicable |
| Luciano César Pontes de Azevedo | none | | | Not applicable |
| Maicon Falavigna | none | | | Not applicable |
| Miriam Cristine Vahl Machado | none | | | Not applicable |
| Regis Goulart Rosa | none | | | Not applicable |
| Roberto Ceratti Manfro | 5b | | President of the Brazilian Association for Organ Transplantation (Former) | Did not participate in the panel |
| Rosana Reis Nothen | 5b | | Coordinator of the National Transplant System of the Brazilian Ministry of Health (Former) | Did not participate in the panel |
| Suzana Margareth Lobo | none | | | Not applicable |
| Tatiana Helena Rech | none | | | Not applicable |
| Thiago Lisboa | none | | | Not applicable |
| Verônica Colpani | none | | | Not applicable |

Declaration of interests

The declaration of interests was adapted from the World Health Organization’s declaration of interest form. All group members must disclose their potential conflicts of interest on the Declaration of Interest Form below. Participants who had any relevant conflicts of interest associated with one or more questions of the document would not be able to participate in the discussion of these specific subjects, but would be allowed full participation in the discussion of all other subjects, including voting if no consensus was reached. One participant declared to have a conflict of interest relevant to question 5b, as shown in the summary table of conflicts of interest declared by the guideline participants.

**DECLARATION OF INTEREST FORM***

*Document adapted from the World Health Organization’s declaration of interest form.

The Ministry of Health, seeking scientific basis for decisions related to health policies, depends, in many circumstances, on the assistance of external experts who may have interests related to their expertise. To ensure the highest integrity and public confidence in its activities, the Ministry of Health requires that these experts disclose any circumstances that could give rise to a potential conflict of interest related to the subject of the activity in which they will be involved.

Within this context, we would like to invite you to complete the form below, which will help you disclose any financial, professional or other interest that may affect your participation as a member of the drafting group of the **Guidelines for Maintenance of Potential Multiple Organ Deceased Donors**. The form will be submitted to the Guideline’s coordinating team.

**NAME:**

**INSTITUTION:**

**EMAIL**:

Date and title of the meeting or guideline in which you are involved, including description of subject matter to be considered:

Date: ___________________

__________________________________________________________________________________________________________________________________________________________

Please answer each of the questions below. If the answer to any of the questions is "YES", describe the circumstances on the last page of the form.

The term "you" refers to yourself and your immediate family members (i.e., spouse or partner with whom you have a similar close personal relationship, and your children). "Commercial entity" includes any commercial business, an industry association, research institution or other enterprise whose funding is significantly derived from commercial sources with an interest related to the subject of the meeting or of this guideline. "Organization" includes a governmental, international or non-profit organization. "Meeting" includes a series or cycle of meetings.

1. **EMPLOYMENT AND CONSULTING**

Within the past 4 years, have you received remuneration from a commercial entity or other organization with an interest related to the subject of the meeting or of this guideline?

| 1a | Employment | Yes ( ) | No ( ) |
| --- | --- | --- | --- |
|  |  |  |  |
| 1b | Consulting, including service as a technical or other advisor | Yes ( ) | No ( ) |

1. **RESEARCH SUPPORT**

Within the past 4 years, have you or has your research unit received support from a commercial entity or other organization with an interest related to the subject of the meeting or of this guideline?

| 2a | Research support, including grants, collaborations, sponsorships, and other funding | Yes ( ) | No ( ) |
| --- | --- | --- | --- |
|  |  |  |  |
| 2b | Non-monetary support valued at more than R$ 1000.00 overall (include equipment, facilities, research assistants, paid travel to meetings, etc.)  Support (including honoraria) for being on a speakers bureau, giving speeches or training for a commercial entity or other organization with an interest related to the subject of the meeting or of this guideline? | Yes ( ) | No ( ) |

1. **INVESTMENT INTERESTS**

Do you have current investments (valued at more than R$ 10 000.00) in a commercial entity with an interest related to the subject of the meeting or of this guideline? Please also include indirect investments, such as a trust or holding company. You may exclude mutual funds, pension funds or similar investments that are broadly diversified and on which you exercise no control.

| 3a | Stocks, bonds, other securities | Yes ( ) | No ( ) |
| --- | --- | --- | --- |
|  |  |  |  |
| 3b | Commercial business interests (e.g., proprietorships, partnerships, joint ventures, board memberships) | Yes ( ) | No ( ) |

1. **INTELLECTUAL PROPERTY**

Do you have any intellectual property rights that might be enhanced or diminished by the outcome of the meeting or of this guideline?

| 4a | Patents, trademarks, or copyrights (including pending applications) | Yes ( ) | No ( ) |
| --- | --- | --- | --- |
| 4b | Proprietary know-how in a substance, technology or process | Yes ( ) | No ( ) |

1. **PUBLIC STATEMENTS AND POSITIONS (DURING THE PAST 3 YEARS)**

| 5a | As part of a regulatory, legislative or judicial process, have you provided an expert opinion or testimony, related to the subject of the meeting or of this guideline, for a commercial entity or other organization? | Yes ( ) | No ( ) |
| --- | --- | --- | --- |
|  |  |  |  |
| 5b | Have you held an office or other position, paid or unpaid, where you represented interests or defended a position related to the subject of the meeting or of this guideline? | Yes ( ) | No ( ) |

1. **ADDITIONAL INFORMATION**

| 6a | If not already disclosed above, have you worked for the competitor of a product that is the subject of the meeting or of this guideline, or will your participation in the meeting or this guideline enable you to obtain access to a competitor’s confidential proprietary information, or create for you a personal, professional, financial or business competitive advantage? | Yes ( ) | No ( ) |
| --- | --- | --- | --- |
|  |  |  |  |
| 6b | To your knowledge, would the outcome of the meeting or of this guideline benefit or adversely affect interests of others with whom you have substantial common personal, professional, financial or business interests (such as your adult children or siblings, close professional colleagues, administrative unit or department)? | Yes ( ) | No ( ) |
|  |  |  |  |
| 6c | Excluding the Ministry of Health or Hospital Moinhos de Vento, has any person or entity paid or contributed toward your travel costs related to this meeting or guideline? | Yes ( ) | No ( ) |
|  |  |  |  |
| 6d | Have you received any payments (other than for travel costs) or honoraria for speaking publicly on the subject of this meeting or guideline? | Yes ( ) | No ( ) |
|  |  |  |  |
| 6e | Is there any other present aspects or circumstances not addressed above that might be perceived as affecting your objectivity or independence? | Yes ( ) | No ( ) |

EXPLANATION of "YES” responses: If the answer to any of the above questions is "YES", briefly describe the circumstances on this page (below). If you do not describe the nature of an interest or if you do not provide the amount or value involved, the conflict will be assumed to be significant.

| Numbers 1-4  Type of interest, question number and category (e.g., intellectual property 4.a copyrights) and basic descriptive details | Name of company, organization, or institution | Belongs to you, a family member, employer, research unit or other? | Amount of income or value of interest (if not disclosed, is assumed to be significant) | Current interest (or year ceased) |
| --- | --- | --- | --- | --- |
|  |  |  |  |  |
|  |  |  |  |  |
| Numbers 5-6: Describe the subject, specific circumstances, parties involved, time frame and other relevant details | | | | |

**CONSENT TO DISCLOSURE**

By completing and signing this form, you consent to the disclosure of any relevant conflicts of interest to other meeting participants and in the resulting guideline or product.

**DECLARATION**

I hereby declare on my honor that the disclosed information is true and complete to the best of my knowledge.

Should there be any change to the above information, I will promptly notify the responsible staff of this guideline and complete a new declaration of interest form that describes the changes. This includes any change that occurs before or during the meeting or the guideline itself and through the period up to the publication of the final results or completion of the activity concerned.

Date: ________________ Signature: _______________________________
