# Supplementary material for: Brazilian guidelines for the management of brain-dead potential organ donors. The task force of the AMIB, ABTO, BRICNet, and the General Coordination of the National Transplant System
Source: Ann Intensive Care. 2020 Dec 14;10:169. doi: 10.1186/s13613-020-00787-0 (PMC7736434; doi:10.1186/s13613-020-00787-0)
Supplement: Supplementary file 3 — Additional file 3. Checklist for clinical management of brain-dead potential organ donor. [file 13613_2020_787_MOESM3_ESM.docx]

**Additional file 3.** Evidence-based bed-side checklist for clinical management of brain-dead potential organ donors.

| **Name: _______________________________________________________**  **Date and time of 1^st^ clinical examination consistent with brain death: _____/_____/________ _____:_____**  **Current date and time: _____/_____/________ _____:_____** | | | | |  |
| --- | --- | --- | --- | --- | --- |
| **GOALS TO BE ACHIEVED** | **STATUS** | **IMMEDIATE ACTIONS WHEN STATUS = "NO"** | | **ACTION TAKEN?** | |
| SaO_2_ ≥ 90%? | **□** Yes **□** No **□** NA | Adjust FiO_2_ and/or PEEP to SaO_2_ ≥ 90% | | **□** Yes **□** No | |
| Vt of 6 to 8 mL/kg of predicted weight? | **□** Yes **□** No **□** NA | Adjust Vt to 6 to 8 mL/kg | | **□** Yes **□** No | |
| PEEP ≥ 8 cm H_2_O? | **□** Yes **□** No **□** NA | Adjust PEEP to ≥ 8 cm H_2_O | | **□** Yes **□** No | |
| MAP ≥ 65 mm Hg and good tissue perfusion after a crystalloid bolus? | **□** Yes **□** No **□** NA | Continue fluid infusion while there is volume responsiveness (ex.: ∆Pp ≥ 13% / ∆MAP ≥ 8% / ∆SV ≥ 10% / CVP < 8 mm Hg) | | **□** Yes **□** No | |
| MAP ≥ 65 mm Hg and good tissue perfusion after volume adjustment? | **□** Yes **□** No **□** NA | Maintain / initiate norepinephrine (dopamine if bradycardia) | | **□** Yes **□** No | |
| Vasopressin and hydrocortisone were associated after maintaining / initiating norepinephrine / dopamine? | **□** Yes **□** No **□** NA | Add vasopressin (1 IU bolus + 0.5-2.4 IU / h) and  Add hydrocortisone 100 mg 8/8 h | | **□** Yes **□** No  **□** Yes **□** No | |
| Diuresis (urine output) < 4 mL/kg/h? | **□** Yes **□** No **□** NA | Assess need for volume replacement  Maintain / initiate vasopressin or desmopressin (IV) | | **□** Yes **□** No | |
| Na^+^ < 155 mEq/L? | **□** Yes **□** No **□** NA | Correct and order laboratory control in 6 h | | **□** Yes **□** No | |
| K^+^ between 3.5 and 5.5 mEq/L? | **□** Yes **□** No **□** NA | Correct and order laboratory control in 6 h | | **□** Yes **□** No | |
| Mg^++^ > 1.6 mEq/L? | **□** Yes **□** No **□** NA | Correct and order laboratory control in 6 h | | **□** Yes **□** No | |
| Capillary glycemia < 180 mg/dL? | **□** Yes **□** No **□** NA | Insulin IV to maintain glycemia between 140 and 180 mg/dL | | **□** Yes **□** No | |
| Hemoglobin ≥ 7 g/dL? | **□** Yes **□** No **□** NA | Transfuse red blood cells to Hb ≥ 7g/dL | | **□** Yes **□** No | |
| Absence of infection? | **□** Yes **□** No **□** NA | Initiate / maintain antibiotic therapy | | **□** Yes **□** No | |
| Proper body temperature?  - No vasopressor: Goal: 34-35^o^C (after clinical tests)  - With vasopressor: > 35^o^C | **□** Yes **□** No **□** NA | Get 34 to 35^o^C if without vasopressor  Get > 35^o^C if with vasopressor | **□** NA **□** Yes **□** No  **□** NA **□** Yes **□** No | | |
| **Nurse:___________________________________________________Intensivist:___________________________________________** | | | |  | |

CVP, central venous pressure; ΔPp, pulse pressure respiratory variation; ΔSV, stroke volume respiratory variation; FiO_2_, fraction of inspired oxygen; Hb, hemoglobin; K+, potassium; MAP, mean arterial pressure; Mg++, magnesium; Na+, sodium; PEEP, positive end-expiratory pressure; SaO_2_, arterial oxygen saturation; Vt, tidal volume.
